# The effects of hyperbaric oxygen therapy (HBOT) on coronavirus disease-2019 (COVID-19): a systematic review

**DOI:** 10.1186/s40001-021-00570-2

**Published:** 2021-08-19

**Authors:** Shahram Oliaei, SeyedAhmad SeyedAlinaghi, Mohammad Mehrtak, Amirali Karimi, Tayebeh Noori, Pegah Mirzapour, Alireza Shojaei, Mehrzad MohsseniPour, Seyed Peyman Mirghaderi, Sanam Alilou, Parnian Shobeiri, Hadiseh Azadi Cheshmekabodi, Esmaeil Mehraeen, Omid Dadras

**Affiliations:** 1 HBOT Research Center, Golestan Hospital, Islamic Republic of Iran, Navy and AJA Medical University, Tehran, Iran; 2 Iranian Research Center for HIV/AIDS, Iranian Institute for Reduction of High Risk Behaviors, Tehran University of Medical Sciences, Tehran, Iran; 3 Healthcare Services Management, School of Medicine and Allied Medical Sciences, Ardabil University of Medical Sciences, Ardabil, Iran; 4 School of Medicine, Tehran University of Medical Sciences, Tehran, Iran; 5 Department of Health Information Technology, Zabol University of Medical Sciences, Zabol, Iran; 6 Health Information Technology, School of Health Information Management and Information Sciences, Iran University of Medical Sciences, Tehran, Iran; 7 AMAD Research Institute, Supreme National Defense University, Tehran, Iran; 8 Department of Health Information Technology, Khalkhal University of Medical Sciences, 1419733141 Khalkhal, Iran; 9 Department of Global Health and Socioepidemiology, Graduate School of Medicine, Kyoto University, Kyoto, Japan

**Keywords:** COVID-19, Hyperbaric oxygenation, Hyperbaric oxygen therapy, SARS-CoV-2

## Abstract

**Background:**

Oxygenation serves as a cornerstone in the treatment of COVID-19, and several methods have been extensively studied so far. Herein, we aimed to systematically review the studies discussing hyperbaric oxygen therapy (HBOT) to examine its reported efficacy and adverse events in patients with COVID-19.

**Methods:**

We systematically searched and retrieved the relevant articles using keywords on the online databases, including PubMed, Scopus, Embase, Web of Science, and Cochrane databases up to April 11th, 2021. The retrieved records underwent a two-step title/abstract and full-text screening process, and the eligible papers were identified. National Institutes of health (NIH) quality assessment tool was used for this study. This study was registered in the International Prospective Register of Systematic Reviews (PROSPERO) with ID CRD42021269821.

**Results:**

Eight articles from three countries were included. All the included studies had good and fair quality scores, with no poor studies included in this systematic review (Good: *n* = 5, Fair: *n* = 3). Studies were divided into clinical trials and case reports/series. Most of the studies used HBOT less than 1.5–2 absolute atmospheres (ATA) for 90 min sessions and thereafter sessions were decreased to 60 min. Trials demonstrated most of the patients recovered after receiving HBOT, and blood oxygen saturation increased after several sessions of HBOT.

**Conclusion:**

Overall, HBOT seems to be a safe and effective oxygenation method in patients with COVID-19. However, there is limited knowledge and evidence regarding the effects and mechanism of HBOT in COVID-19 treatment, and further evaluations require extensive well-designed studies.

## Background

COVID-19 is an acute respiratory infection caused by the SARS-CoV-2; it emerged as a novel human pathogen in China at the end of 2019 continues to be a pandemic worldwide [1–4]. The most common manifestations are pneumonia, high fever, myalgia, dry cough, and chest pain [5–9]. The death rate due to COVID-19 varies from 1% to more than 7%, and respiratory failure is the main cause [10, 11]. Research is underway to identify and evaluate the effectiveness and safety of interventions to treat the patients with COVID-19 based on their disease severity [12, 13].

Approximately, 15–20% of hospitalized patients present with hypoxemic respiratory failure, accompanied by the need for oxygen supplementation [14]. Hyperbaric oxygen therapy (HBOT) has been proposed as an alternative therapeutic approach to address COVID-19-associated hypoxia [12, 15]. HBOT is recognized as an effective treatment for replacing any form of oxygen deficiency [14]. HBOT is a non-invasive treatment and serves as primary or adjunctive therapy in various medical conditions [16]. The efficacy of HBOT has been documented in several systemic illnesses, such as arterial gas embolism, carbon monoxide poisoning, decompression sickness, crush injuries, and diabetic foot ulcer [17].

HBOT involves intermittently using high concentration oxygen (100%) in an environmental pressure higher than one absolute atmosphere (atm) inside a chamber to enhance the amount of oxygen dissolved in the body’s tissues [18, 19]. HBOT can increase the circulation and delivery of oxygen under high pressure, making the tissue uptake more efficient and improve hypoxia in COVID-19 patients [17]. In addition, hyperoxygenation of arterial blood with plasma-dissolved oxygen during HBOT has a strong anti-inflammatory effect and may have a direct virucidal impact on COVID-19 [20]. Preliminary clinical evidence of HBOT treatment in hypoxemic COVID-19 patients demonstrated clinical improvement, e.g., reduce ICU admission and prevent transition to mechanical ventilation [15, 21].

HBOT is regarded as a safe and low-risk intervention [22]. There are no contraindication to the use of HBOT in patients with viral, bacterial or fungal infections [23]. The only major contraindications to HBOT are untreated pneumothorax and respiratory failure requiring mechanical ventilation [20]. Recent studies pointed out that HBOT could be a decisive treatment for improving outcomes in patients with COVID-19 pneumonia, especially at early stages, and it could also be beneficial during the intubation period [17]. The objective of this study is to review and discuss the efficacy and adverse events of HBOT in patients with COVID-19.

## Methods

### Design

We systematically searched and retrieved the relevant articles using keywords on the online databases, including PubMed, Scopus, Embase, Web of Science, and Cochrane databases up to April 11th, 2021. We reviewed the retrieved articles and removed the duplicates. The remaining records underwent a two-step screening process. First, a researcher (A.S.) screened the records based on their title/abstract, and irrelevant records were excluded. Then, A.S. examined the full-text of the remaining documents based on their cohesion to inclusion criteria, and the eligible studies were identified. Another researcher (A.K.) addressed any uncertainty during the review process. This study was registered in the International Prospective Register of Systematic Reviews (PROSPERO) with ID CRD42021269821.

### Search strategy

We performed a systematic search on the online databases using the keywords such as “Hyperbaric oxygen therapy” and “COVID-19”.

### Inclusion/exclusion criteria

We included all articles, including case reports, case series, clinical trials, cross-sectional, case–control, and cohort studies investigating the effect of HBOT on COVID-19 outcomes; and therefore, the exclusion criteria were as follows:

1. Non-original studies, including review articles, meta-analyses, and non-original editorials
2. Unavailability of full texts and abstracts/conference abstracts
3. Ongoing clinical trials with unpublished results.

### Data acquisition

A researcher designed the data extraction table. Three other researchers extracted the data related to the patients’ characteristics, country of origin, clinical manifestation, laboratory findings, time/pressure/duration of HBOT, patients’ outcome were extracted and included in the abovementioned table. Another researcher checked the data and addressed the controversies.

### Quality assessment

We utilized the National Institutes of Health (NIH) quality assessment tool [24] to evaluate the included studies. We used the scores of 7–9, 4–6, and 0–3 to represent good, fair, and poor ratings for the case series and case reports. For cross-sectional cohort and controlled interventional studies, 11–14, 6–10, and 0–5 were utilized, respectively. We chose the scores of 9–12, 5–8, and 0–4 for case–controls (Table 1).

**Table 1:** Applying the NIH quality assessment tool for all the included studies

Study	Total score	Quality rating (good, fair, or poor)	Study	Total score	Quality rating (good, fair, or poor)
Controlled interventional studies (score out of 14)					
Gorenstein, S. A	11	Good	Petrikov, S. S	8	Good
Levina, O. A	10	Fair			
Case series and case reports (score out of 9)					
Chen, R. Y	6	Fair	Guo, D. Z	6	Fair
Liang, Y	7	Good	Thibodeaux, K	7	Good
Xiao-Ling, Z	7	Good			

## Results

**Fig. 1:**
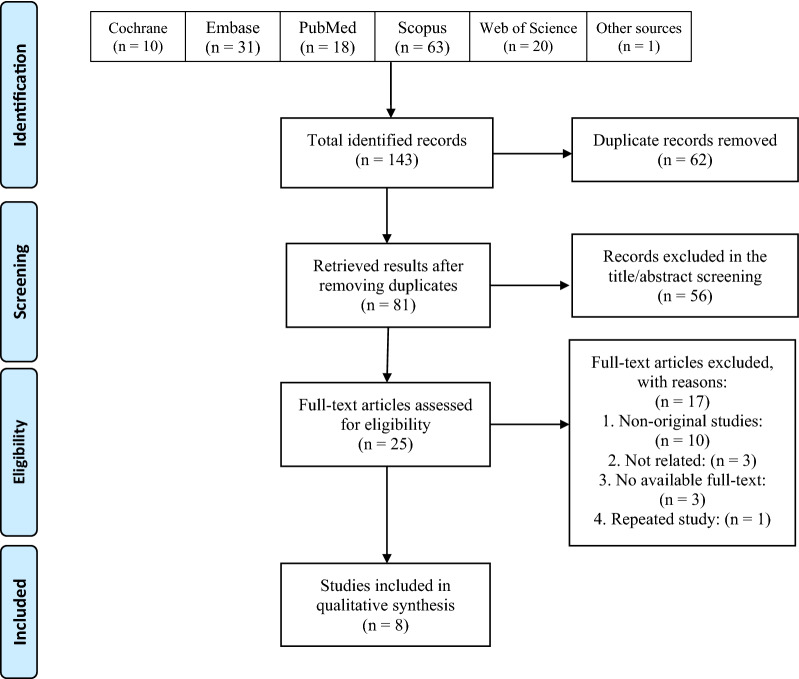
PRISMA flow diagram of the study selection process

We systematically searched the databases mentioned above, and this search yielded 143 results. Of these, 56 were duplicate records. Of the remaining, 64 were removed in the title/abstract screening, and 25 articles entered the full-text screening process, of which 8 articles were finally met the eligibility criteria and included in this review. Figure 1 illustrates the details of the selection process. Three studies were clinical trials, and the other five studies were either case series or case reports. These eight studies are from three countries of China, USA, and Russia.

All the included studies had good and fair quality scores. No poor quality study was included in this review (Good: *n* = 5, Fair: *n* = 3) (Table 1). Studies were conducted in different countries, including Russia, China, and USA. Studies were divided into clinical trials and case series. Eight studies were supporting the use of HBOT in patients infected with SARS-CoV-2. Trials showed most of the patients recovered after receiving HBOT, and blood oxygen saturation increased after several sessions of HBOT. Although one study did not support the hypothesis of the present review, other studies suggested that HBOT could be an effective measure to correct the hypoxemia induced by COVID-19 (Table 2). Of the studies, 3 clinical trials were trying to show the efficacy of the HBOT on patients infected with COVID-19. The results indicated a significant effect of HBOT on patients, and it was safe and beneficial for them to breathe 100% oxygen [14, 25, 26]. Treatment adverse events were very limited and Gorenstein et al. reported mild epistaxis not related to HBOT, ear pain, and claustrophobia [25], but the studies are few and lack large groups of patients, and therefore, adverse events should be further studied in larger clinical trials.

**Table 2:** Details of the data presented by the included studies

ID	First author (reference)	Type of study	Country	Study population	Age (year)	Male	Comorbidities	Pressure and time per day	Baseline laboratory values	Patients’ outcome	Conclusion
1	Chen [27]	Case series	China	*N* = 5 Chest CT showed typical pulmonary imaging changes of COVID-19, and nucleic acid tests of SARS-CoV-2 were positive	24–69 (mean 47.6)	Male 80%	Hypertension, coronary heart disease, acute myocardial infarction, and the coronary stent implantation	2.0 ATA for Patient 1# 1.6ATA for the other patients 90 min in first Treatment & 60 min in the followed	Lymphocyte count and LYM%: (0.61 ± 0·35 × 10^9^/L before vs. 1.09 ± 0·24 × 10^9^/L after, *P* < 0.05) Blood CRP (before): 30.56 ± 1.15 mg/L (After):3.98 ± 1.50 mg/L WBC Before: 6.78 ± 0.39 × 10^9^/L After: 5.64 ± 1.40 × 10^9^/L	All the symptoms were basically relieved except for mild breathlessness (motion) complained by every patient The mean value of daily SpO_2_ of Patient 1# was restored 95% after 5 days, while 2# and 3# after 3 days, 4# after 2 days, and 5# after 1 day	HBOT can dramatically increase the amount of dissolved oxygen in the blood and treatment to critically ill COVID-19 patients
2	Gorenstein [25]	Clinical trial	USA	1) Hyperbaric oxygen therapy, (*N* = 20) 18 years and older, confirmed COVID-19, SpO_2_ < 93% on room air 2) Controls (Propensity-matches patients), (*N* = 60)	1) 58.4 (2) 60.9	1) Male: 18 (90%) 2) Male: 55 (92%)	NA	2 ATA, 90 min	Positive Troponin: 1) 0%, 2) 0% All the following are mean and none are significantly different (units not reported): D-dimer: 1) 1142, 2) 1870 Ferritin: 1) 1490, 2) 1382 CRP: 1) 120, 2) 137 LDH: 1) 496, 2) 475	Mechanical ventilation: 1) *n* = 2 (10%), 2) 18 (30%) Death: 1) *n* = 2 (10%), 2) 13 (21.7%)	The adjusted hazard ratio for time to death = 0.37 (95% CI of 0.10 to 1.37) The adjusted hazard ratio for time to mechanical ventilation = 0.37 (95% CI of 0.10 to 1.37) Few adverse events occurred in the form of epistaxis (not related to HBOT), ear pain, and claustrophobia, and all were classified as mild One severe case of hypoxic arrest that was concluded as not related to HBOT
3	Guo [28]	Case report	China	Symptoms of both cases: shortness of breath; respiratory rate (RR) ≥ 30 breaths/min; finger pulse oxygen saturation (SpO_2_) ≤ 93% at rest; and oxygen index (P/F ratio: PaO_2_/FiO_2_ ≤ 300 mmHg.*N* = 2	Case 1) 57 Case 2) 64	Case 1) male Case 2) male	Case 1) Cough, fever, fatigue, and hypertension Case 2) Cough, fever, diabetes, and coronary heart disease	1.5 atmospheres absolute HBO_2_ with an oxygen concentration of more than 95% for 60 min per treatment, once a day for one week	NA	For both patients, dyspnea and shortness of breath were immediately alleviated after the first HBO_2_ treatment. The RR also decreased daily. The decreasing trend of SO_2_ and P/F ratio was immediately reversed and increased day by day. Ratio corresponding to immune function gradually recovered. D-dimer corresponding to peripheral circulation disorders and serum cholinesterase, reflecting liver function had improved. Follow-up chest CT showed that the pulmonary inflammation had clearly subsided	Hyperbaric oxygen therapy treatment may rapidly improve the progressive hypoxemia of patients with COVID-19 pneumonia
4	Levina [14]	Clinical trial	Russia	1) 10 patients with the diagnosis “Coronavirus infection caused by the virus SARS-CoV-2” (moderately severe patients) 2) 22 patients with the diagnosis “Coronavirus infection caused by the virus SARS-CoV-2” in serious condition	1) 63.5 [51; 74] 2) 59 [51,75; 67]	1) Male: 5 (50.0%) 2) Male: 10 (45.5%)	Claustrophobia ear pain	1.4–1.6 ATA for no more than 60 min	Not available	The patients showed an increase in blood oxygen saturation in patients in both surveyed groups, as well as positive dynamics in the form of a decrease in shortness of breath, an improvement in general well-being	Inclusion of daily sessions (at least 4) of excessive oxygen delivery in "soft" states (1.4–1.6 ATA) in the complex treatment of COVID-19, safety, and its initial positive effect on the mental state of the examined patients and the dynamics of blood oxygen saturation
5	Liang [29]	Case report	China	Chills and a body temperature of 37.8 °C Chest CT examination multiple patchy ground-glass opacity (GGO) shadows in the S I and S II segments of the upper lobe of the right lung and the S I + II segment of the upper lobe of the left lung. *N* = 1	69	Male	A history of coronary atherosclerotic heart disease and underwent coronary stent implantation	Total daily oxygen inhalation time of 95 min and an oxygen dose of 216 unit of pulmonary toxic dose (UPTD)	White blood cell counts 3.68 × 109/L Lymphocyte count 1.47 × 109/L C-reactive protein 22.4 mg/L D-dimer 0.28 mg/L Prothrombin time 11.3 s Partial thromboplastin activation time 28.2 s	Blood gas examination showed a PO_2_ of 122 mmHg, PCO_2_ of 37.3 mmHg, and SO_2_ reaching 99%. Re-examination of CT showed that the area of consolidation in bilateral lungs decreased. The patient was switched to a nasal catheter for oxygen inhalation. One month after discharge, follow-up CT examination showed diffuse GGO shadows in both lungs, while the consolidation shadows and fibrous cord shadows completely resolved	Hyperbaric oxygen therapy may directly cause persistent accumulation of oxygen debt under hypoxic tissues and organs throughout the body and subsequent damage to important oxygen-consuming tissues and organs, providing a good systemic functional basis for the body to combat viral infections
6	Petrikov [26]	Clinical trial	Russia	1) Study group, (*N* = 57), 18 years and older, confirmed COVID-19, SpO_2_ = 91.3 ± 5.9% 2) Control group (*N* = 30)	1) 58.8 ± 13.6 2) 64.5 ± 12.7	1)Male: 30 (52.6%) 2) Male: 13 (43.3%)	NA	1.4_1.6 ATA mode for 40 min	Blood Malondialdehyde: 1) decrease from 4.34 ± 0.52 µmol/L to 3.98 ± 0.48 µmol/L	NA	The use of Hyperbaric oxygen therapy increases the effectiveness of treatment
7	Thibodeaux [21]	Case series	USA	Study group, (*N* = 5), 18 years and older, confirmed COVID-19	39–63	1) 1 (20%)	Obesity (80%) Diabetes (60%) Hypertension (80%) Viral pneumonia (40%)	2.0 ATA for 90 min	D-dimer (pre): 1 = 12,070 mg/mL D-dimer (post): 1 = 4324 mg/mL	Recovered: *n* = 5 (100%) oxygen saturation of 97% on 45% FiO_2_ Oxygen saturation(pre): 95.5 ± 2.61% Oxygen saturation(post): 94.6 ± 2.30% Respiratory rate, breaths/min (pre): 35.4 ± 8.47 Respiratory rate, breaths/min (post): 28 ± 7.55	HBOT can reduce the need for mechanical ventilation, and increase oxygen saturation in patients with COVID-19
8	Xiao-Ling [30]	Case report	China	*N* = 1 coronavirus disease 2019 (COVID-19) patient with endotracheal intubation	87	Male	NA	NA	NA	After four times HBOT: 1. Improved function of liver and kidney 2. Improved blood coagulation	HBO_2_ significantly reduces CO_2_ retention in COVID-19 patients

*CRP* C-reactive protein, *LDH* Lactate dehydrogenase, *ATA* Absolute atmosphere, *NA* Not Available

## Discussion

Aggressive oxygen therapy is a mainstay treatment for critically ill COVID-19 patients and has been used in various methods to reduce mortality [31]. For severe COVID-19 patients with acute hypoxemic respiratory failure, in addition to conventional oxygen therapy, the National Institutes of Health (NIH) guideline [32] suggests high-flow nasal cannula (HFNC) oxygen for the treatment as the first choice. As a second choice, non-invasive positive pressure ventilation (NIPPV) could be applied under close monitoring. Further refractory hypoxemia necessitates endotracheal intubation in these patients. At last, extracorporeal membrane oxygenation (ECMO), however, with inconclusive evidence, could potentially use as rescue therapy for patients with severe acute respiratory distress syndrome (ARDS) [33, 34]. The whole aim of extra oxygenation is to reach O_2_ saturation of 92–96% [32, 35–37], which is attained in some studies included in this review [14, 21, 26, 27, 38]. HBOT, regardless of the promising evidence on respiratory improvement, has not yet been recommended in the guidelines for severe COVID-19 patients.

HBOT benefits patients by intensifying the oxygen pressure in the alveoli. Consequently, the diffusion rate and the diffusion instance of oxygen will increase compared to standard oxygen therapy (e.g., face mask, invasive ventilation, non-invasive ventilation, nasal cannula, and ECMO) [39]. HBOT provides tissue perfusion exchange capacity due to the increased diffusion instance of oxygen, distinguishing HBOT from all other oxygen therapy methods. Patients treated with HBOT showed improvements in their clinical factors and indexes as follows: (1) arterial blood gas analysis, (2) liver function tests, (3) complete blood count (CBC, diff), and (4) improvement of lung structure clearance based on computed tomography (CT-scan) [39].

It should be noted that COVID-19 patients in hyperbaric chambers need special monitoring and considerations. Briefly, electrocardiogram, pulse oximetry, and temperature monitoring are the backbones of monitoring these patients. The risk of fire accidents increases as high-pressure oxygen is used in a chamber consisting of automated external defibrillator (AED) paddles and external defibrillators; thus, fire prevention should be considered. The ICU personnel should consistently check the endotracheal tube cuff pressure in an intubated patient. Last but not least, as a general rule, personal protective equipment (PPE) must be a priority for health workers caring for such patients [39].

HBOT is currently indicated in conditions such as gas embolism, CO_2_ and cyanide poisoning, severe anemia, and other pathologic situations [40]. In the HBOT, patients breathe 100% pure oxygen with high pressure. It may have a beneficial effect in all stages of cell oxygenation, from lung function and alveolar oxygen exchange to the hemoglobin capacity and oxygen delivery to the tissues [27]. In the included studies, we can perceive the merits of HBOT treatments to hypoxemia in critically ill COVID-19 patients. However, further randomized clinical trials with larger sample sizes are necessary to attain compelling evidence to establish HBOT as an effective treatment option in guidelines.

All of the included studies showed promising outcomes in COVID-19 patients who underwent HBOT. This type of oxygen delivery resolved severe COVID-19 symptoms and boosted the general well-being of treated patients, along with correcting hypoxia and elevating O_2_ saturation. While in all manuscripts, HBOT reduced mortality, only one clinical trial reported a mortality rate of 10% (2 out of 20) in COVID-19 patients undergoing HBOT vs. 22% of that in controls [25]. No death was reported in the rest of the studies; this may be due to the limited study population size and study design, highlighting the need for further clinical trials with larger sample sizes to justify this treatment’s potential benefits and side effects.

HBOT remains one of the most effective and safest interventions to compensate for oxygen deprivation in acute respiratory distress syndrome (ARDS) [17]. Since patients breathe naturally in HBOT chambers, they would probably not experience any serious side effects during major respiratory interventions such as mechanical ventilation. Complications such as middle ear and pulmonary barotrauma, oxygen toxicity (mainly involving central nervous system), and ocular effects were reported in previous studies using HBOT for other health issues, with applied pressure mostly exceeding 2.0 ATA [41]. However, some of these adverse effects were not documented in the included studies in this review, probably due to the maximum of 2 ATA pressure, smaller sample size, and different study designs. Only Gorenstein et al. reported mild cases of epistaxis, ear pain, and claustrophobia [25]. One major shortcoming of HBOT might be the lack of accessibility, as there are even shortages in the availability of standard oxygen delivery and HBOT is probably also less available in many centers.

### Limitations

Although this study is the first endeavor that systematically explored the effect of HBOT on severe respiratory manifestation in COVID-19 patients, some limitations weaken the strength of retrieved evidence, mainly due to the paucity and the design of the selected studies. Data from case series and case reports face bias due to the absence of a control group and limited population enrolled in the study. In addition, many studies were held on critically ill patients who are refractory to routine oxygen therapy; consequently, the authors cannot generalize these data from our sample to the general population of COVID-19 patients. We attempted to include patients only once in our review, but some case series may represent duplicate data that is shared with another case study included from the same setting. In addition, some articles were not written in English, and therefore, we had difficulty translating to perceive the correct concept of it. Last but not least, HBOT equipment are not available in many centers, and this shortage is also more prominent in the pandemic where the availability of such facilities are facing challenges. Therefore, limited patients have access to this treatment option and the populations studied in this systematic review may not be representative of healthcare facilities.

## Conclusion

Overall, HBOT seems to be a safe and effective method of oxygenation in patients with COVID-19. However, its large space occupation and lack of availability in large numbers may limit its use in the settings of a pandemic where many patients require oxygenation, and this shortcoming needs to be addressed. There is limited knowledge and evidence regarding the effects of HBOT in the settings of COVID-19, and further well-designed trials with larger sample sizes are recommended to carefully assess the outcomes of this treatment modality and compare it with other oxygenation methods.

## Data Availability

The authors stated that all information provided in this article could be shared.
